# Specific Absorption Rate Dependency on the Co^2+^ Distribution and Magnetic Properties in Co_x_Mn_1-x_Fe_2_O_4_ Nanoparticles

**DOI:** 10.3390/nano11051231

**Published:** 2021-05-07

**Authors:** Venkatesha Narayanaswamy, Imaddin A. Al-Omari, Aleksandr S. Kamzin, Bashar Issa, Huseyin O. Tekin, Hafsa Khourshid, Hemant Kumar, Ambresh Mallya, Sangaraju Sambasivam, Ihab M. Obaidat

**Affiliations:** 1 Department of Physics, United Arab Emirates University, Al-Ain 15551, United Arab Emirates; venkateshnrn@gmail.com (V.N.); sambaphy@gmail.com (S.S.); 2 Department of Physics, Sultan Qaboos University, P.O. Box 36, Muscat PC 123, Oman; ialomari@squ.edu.om; 3 Ioffe Physical Technical Institute, 194021 St. Petersburg, Russia; askam@mail.ioffe.ru; 4 Department of Medical Diagnostic Imaging, College of Health Sciences, University of Sharjah, Sharjah 27272, United Arab Emirates; bissa@sharjah.ac.ae (B.I.); htekin@sharjah.ac.ae (H.O.T.); 5 Department of Physics, College of Sciences, University of Sharjah, Sharjah 27272, United Arab Emirates; hkhurshid@sharjah.ac.ae; 6 Materials Engineering, Indian Institute of Science, Bangalore 560012, India; hemantkumar2@iisc.ac.in (H.K.); ambreshm@iisc.ac.in (A.M.)

**Keywords:** specific absorption rate, cobalt ferrite nanoparticles, co-precipitation, magnetization

## Abstract

Mixed ferrite nanoparticles with compositions Co_x_Mn_1-x_Fe_2_O_4_ (*x* = 0, 0.2, 0.4, 0.6, 0.8, and 1.0) were synthesized by a simple chemical co-precipitation method. The structure and morphology of the nanoparticles were obtained by X-ray diffraction (XRD), transmission electron microscope (TEM), Raman spectroscopy, and Mössbauer spectroscopy. The average crystallite sizes decreased with increasing *x*, starting with 34.9 ± 0.6 nm for MnFe_2_O_4_ (*x* = 0) and ending with 15.0 ± 0.3 nm for CoFe_2_O_4_ (*x* = 1.0). TEM images show an edge morphology with the majority of the particles having cubic geometry and wide size distributions. The mixed ferrite and CoFe_2_O_4_ nanoparticles have an inverse spinel structure indicated by the splitting of A_1g_ peak at around 620 cm^−1^ in Raman spectra. The intensity ratios of the A_1g_(1) and A_1g_(2) peaks indicate significant redistribution of Co^2+^ and Fe^3+^ cations among tetrahedral and octahedral sites in the mixed ferrite nanoparticles. Magnetic hysterics loops show that all the particles possess significant remnant magnetization and coercivity at room temperature. The mass-normalized saturation magnetization is highest for the composition with *x* = 0.8 (67.63 emu/g), while CoFe_2_O_4_ has a value of 65.19 emu/g. The nanoparticles were PEG (poly ethylene glycol) coated and examined for the magneto thermic heating ability using alternating magnetic field. Heating profiles with frequencies of 333.45, 349.20, 390.15, 491.10, 634.45, and 765.95 kHz and 200, 250, 300, and 350 G field amplitudes were obtained. The composition with *x* = 0.2 (Co_0.2_Mn_0.8_Fe_2_O_4_) with saturation magnetization 57.41 emu/g shows the highest specific absorption rate (SAR) value of 190.61 W/g for 10 mg/mL water dispersions at a frequency of 765.95 kHz and 350 G field strength. The SAR values for the mixed ferrite and CoFe_2_O_4_ nanoparticles increase with increasing concentration of particle dispersions, whereas for MnFe_2_O_4_, nanoparticles decrease with increasing the concentration of particle dispersions. SARs obtained for Co_0.2_Mn_0.8_Fe_2_O_4_ and CoFe_2_O_4_ nanoparticles fixed in agar ferrogel dispersions at frequency of 765.95 kHz and 350 G field strength are 140.35 and 67.60 W/g, respectively. This study shows the importance of optimizing the occupancy of Co^2+^ among tetrahedral and octahedral sites of the spinel system, concentration of the magnetic nanoparticle dispersions, and viscosity of the surrounding medium on the magnetic properties and heating efficiencies.

## 1. Introduction

Advances in the synthesis of magnetic nanoparticles (MNPs) have led to major improvements in various biomedical applications [1]. MNPs are intensely investigated in the fields of drug delivery, MRI contrast agents, and magnetic particle imaging (MPI) [2,3,4]. MNPs produce thermal heating when exposed to an alternating magnetic field (AMF) [5]. If MNPs are localized at the sites of the targeted cancerous cells, subsequent heating will produce no harm to the healthy tissue, causing minimum collateral damage [6]. Ferrite-based nanoparticles are investigated for magnetic hyperthermia (MHT) and, recently, for MPI for dual purposes of imaging and treatment of cancer cells [7]. Various ferrite nanoparticles with core–shell and cubic geometry are subjected to both in vivo and in vitro studies as MRI contrast agents and magnetic MHT agents [8,9]. The crucial requirement for the use of ferrite nanoparticles for MHT is to deal with the post treatment accumulation of nanoparticles in kidney and liver [10]. To address this concern, it is essential to use a minimal dose of nanoparticles to achieve the required temperature of 42–44 °C to kill the cancerous cells. A high specific absorption rate (SAR) is a key feature of MNPs that will lead to dose reduction [11]. SAR is determined by several factors such as the average size, shape, composition, inter-particle interactions, magnetic anisotropy, as well as the frequency and amplitude of the applied alternating magnetic field. MNPs of core–shell geometry and doped ferrite are highly efficient for hyperthermia treatment compared with the pure ferrite phase [12,13,14]. To provide an appropriate thermal dose to the tumor, most current MNPs need a high frequency or high AMF amplitude (H) because of low SAR. The mixed ferrite nanoparticles have shown improved efficacy for magnetic hyperthermia, which can be attributed to the crystallite anisotropy manipulated by the exchange coupling of Mn^2+^, Co^2+^, and Fe^3+^ cations in the spinel lattice with oxygen atoms [15]. Kerroum et al. have reported SAR dependency on field strength in the superparamagnetic nanoparticle system of Zn_x_Fe_3-x_O_4_ (*x* = 0.0–0.5) with particle size of 16 nm, synthesized using the chemical co-precipitation method [16]. The saturation magnetization (M_s_) of nanoparticles was increased up to 120 Am^2^/kg for *x* = 0.3 by homogeneous zinc replacement of iron cations into the magnetite crystallite structure. When *x* was varied between 0 and 0.3, the SAR values increased significantly, but decreased when *x* was less than 0.5. Up to 35 kA/m; the SAR values showed a quadratic dependency on the alternating magnetic field amplitude (H). A strong saturation effect of SAR was observed above this value, which was successfully explained qualitatively and quantitatively by taking into account the non-linear field’s effects and the magnetic field dependence of both Brown and Neel relaxation times.

In the context of linear response theory (LRT), the heating of small superparamagnetic MNPs in small amplitude AMF (such as the Zeeman energy, which is smaller than the thermal energy) is represented by Equation (1) [17]. According to Rosensweig’s model, the magnetization of nanoparticles is proportional to the applied magnetic field, with the proportionality element being the complex susceptibility. In an AMF, the rate of volumetric heat release can be written as given by Equation (1).

(1) $$P={\pi \mu }_{0}\chi^{\prime \prime }H^{2}f$$

where µ_0_ is the vacuum magnetic permeability, f is the frequency, H is the amplitude of the AMF, and χ^″^ the imaginary part of the magnetic susceptibility given by $\chi \;(\chi =\chi^{\prime }-i\chi^{\prime \prime }$) In the LRT, it is assumed that χ stays constant as H increases (M=χH). It is known that this assumption is valid for very small H values. Thus, in the LRT, the heat dissipation of the MNPs has a linear dependence on the AMF frequency and a quadratic dependence on AMF amplitude. The imaginary part of the susceptibility, $\chi^{\prime \prime }$ is given by the following [18]:

(2) $$\chi^{\prime \prime }=\frac{2\pi f\tau }{1+{\left(2\pi f\right)}^{2}}\chi_{0}$$

The static susceptibility ($\chi_{0}$) is given by

(3) $$\chi_{0}=\frac{\mu_{0}M_{s}^{2}V}{k_{B}T}$$

Here, Ms is the saturation magnetization of the material, V is its magnetic volume, k_B_ is the Boltzmann constant, and T is the absolute temperature.

The effective magnetic relaxation time τ is given by

(4) $$\frac{1}{\tau }=\frac{1}{\tau_{N}}+\frac{1}{\tau_{B}}$$

The Brownian relaxation time, $\tau_{B}$, characterizes the particle’s magnetic moment flipping owing to the rotation of the particle itself, and is given by the following [19]:

(5) $$\tau_{B}=\frac{3{\;V}_{H}\;\eta }{k_{B\;}T}$$

where $V_{H}$ is the hydrodynamic volume of the particle and η is the viscosity of the liquid where the particle is immersed. As the Brownian relaxation time as stated in Equation (5) depends on the viscosity of the surrounding medium, the effect becomes more pronounced when heating ability is obtained for ferrogel.

The Néel relaxation time, $\tau_{N}$, is due to the rotation of the magnetic moment of the MNP, and is given by the following [11]:

(6) $$\tau_{N}=\frac{\tau_{o}}{2}\sqrt{\frac{{\pi k}_{B}T}{\mathrm{KV}}}\exp\left(\frac{\mathrm{KV}}{k_{B}T}\right)$$

where K is the magnetic anisotropy of the MNPs and $\tau_{0}$ is a constant ($\approx 10^{-13}-10^{-9}s$). V is the volume of the magnetic core of the particle.

From the magnetic heating mechanism and LRT theory, it can be implied that SAR will vary with the AMF frequency (f), applied field strength (H), and magnetic anisotropy constant (K), which can be tuned by varying the spinel ferrite composition with the doping of Co^2+^, Mn^2+^, Ni^2+^, and Zn^2+^ divalent ions [15]. The distribution of divalent ions and Fe^3+^ ions among tetrahedral and octahedral sites plays an important role in manipulating K. The anisotropy constant (K) is the main deciding factor of Neel relation time as given by Equation (6) at a given temperature. Although heating power has a square dependency on the saturation magnetization, anisotropy constant, and viscosity of the surrounding medium, it still has a crucial role to play in the heating ability of the nanoparticles. It is well established that the inter-particle interactions affect the relaxation time of the magnetic particles and, hence, SAR values [20,21]. The relaxation time is modified by inter-particle interactions via changing the magnetic anisotropy constant and the relaxation time constant. However, the role of inter-particle interactions on the relaxation time is still controversial, where some studies reported an increase in the anisotropy constant and other studies reported the opposite [20,21]. In a recent theoretical study [21], the authors reported an almost linear increase of the anisotropy energy barrier and a quasi-exponential decrease of the relaxation time constant due to inter-particle interactions, which result in a significant decrease in the SAR values in samples with large particle concentrations. Hence, more research is needed to clarify the role of the inter-particle interactions in different experimental conditions.

In the current study, we report the heating ability of the Co_x_Mn_1-x_Fe_2_O_4_ (*x* = 0, 0.2, 0.4, 0.6, 0.8, and 1.0) nanoparticles. The distribution of divalent cation (Co^2+^ and Mn^2+^) and Fe^3+^ among tetrahedral and octahedral sites is thoroughly investigated using Raman spectroscopy. The effect of the composition and concentration of the nanoparticles on the SAR values is studied in detail. To study the effect of the surrounding environment like viscosity of the medium on the heating ability of the nanoparticles, agar ferrogel phantom is used. The heating abilities of the nanoparticles are significantly different for the ferrogels compared with the nanoparticle water dispersions.

## 2. Materials and Methods

### 2.1. Synthesis of Co_x_Mn_1-x_Fe_2_O_4_ (x = 0, 0.2, 0.4, 0.6, 0.8, and 1.0) Nanoparticles

Six sets of Mn^2+^ and Co^2+^ mixed ferrite nanoparticles with compositions Co_x_Mn_1-x_Fe_2_O_4_ (*x* = 0, 0.2, 0.4, 0.6, 0.8, and 1.0) were synthesized using a simple co-precipitation method in aqueous medium. For the synthesis of each batch of nanoparticles, the calculated amount of MnCl_2_, CoCl_2_.6H_2_O, and FeCl_3_ salts was dissolved in 200 mL of deionized water. The solution mixture was preheated to 80 ˚C and 1 N NaOH solution was added drop wise under constant stirring to adjust the pH in the range of 12–13. The solution mixture was heated at 85 ˚C for 1 h and subsequently cooled to room temperature. The nanoparticles synthesized were filtered and then washed with deionized water several times. The synthesized particles were dried under IR lamp and then used for further characterization and magnetic hyperthermia studies. The compositions of the nanoparticles were obtained from SEM-EDS analysis and the values were found to be very similar to those used in the synthesis process (*x* = 0, 0.2, 0.4, 0.6, 0.8, and 1.0). The magnetic hyperthermia studies were conducted for PEG-coated nanoparticles. To coat the nanoparticle with PEG, 500 mg of Co_x_Mn_1-x_Fe_2_O_4_ (*x* = 0, 0.2, 0.4, 0.6, 0.8, and 1.0) nanoparticles were added to 20 mL of solution with a PEG concentration of 2.5 mg/ ml and sonicated for 60 min, then kept at room temperature for 24 h. From this solution, specific concentrations (3, 5, 7, and 10 mg/mL) of nanoparticle dispersions were prepared for the magneto thermal measurements.

### 2.2. Characterization of the Nanoparticles

Structural phases of the nanoparticles and the crystallites sizes were determined from the X-ray diffraction profile using a Shimadzu-6100 powder X-ray diffraction (XRD) diffractometer with Cu-Kα radiation and wavelength 1.542 Å. A 300 keV Titan Themis 300 kV from FEI transmission electron microscope (TEM) was used to obtain bright field images and selected area electron diffraction patterns. The dc magnetic measurements were carried out using a VSM in Physical Properties Measurement System (PPMS) from Quantum Design. Raman spectra was obtained from the nanoparticle pellets using NOST Raman spectrometer consisting of a diode-pumped solid-state laser operating at 532 nm with a charge coupled detector. A standard constant acceleration spectrometer was used in a transmission mode to record the Mössbauer spectra. 57Co (Rd) was used as a radioactive source for this experiment, and the isomer shifts are measured relative to the centroid of α–iron.

### 2.3. Magneto Thermal Measurements

The nanoparticles were dispersed in water by sonication, after which 1 ml dispersions of 3, 5, 7, and 10 mg/mL of each particle concentration were used for obtaining the heating profiles. The heating profiles of nanoparticles were obtained using a nanoScale Biomagnets hyperthermia instrument. The calorimetric measurements were conducted using an AMF, in one set of measurements, where the field strength was fixed at 350 G for all the field frequencies of 765.85, 634.45, 491.10, 390.25, 349.20, and 333.65 kHz. In the second kind of measurement, the field frequency was fixed at 765.85 kHz for all the field strengths of 200, 250, 300, and 350 G. The SAR values for all the concentrations of nanoparticles were evaluated from the slope of the linear part of the heating profile curve according to Equation (7):

(7) $$\mathrm{SAR}\;\left(W/\mathrm{kg}\right)=\frac{C}{m_{\mathrm{MNP}}}\frac{\mathrm{dT}}{\mathrm{dt}}\;$$

where C (J/K) is the heat capacity of the nanoparticle dispersion given by $\;C=c_{\mathrm{MNP}}m_{\mathrm{MNP}}+c_{\mathrm{water}}m_{\mathrm{water}}$, where $c_{\mathrm{MNP}}$, and $c_{\mathrm{water}}$ (J/kg·K) are the specific heat capacities of the MNPs and the water, respectively. $m_{\mathrm{water}}$ is the mass of water and $m_{\mathrm{MNP}}$ (mg) is the mass of MNPs in the nanoparticles in the dispersion. $\frac{\mathrm{dT}}{\mathrm{dt}}$ is the initial slope of the temperature versus time plot. This choice was considered because, at the initial stage of heating, heat transfer between the sample and the environment will be negligible, and thus adiabatic conditions are valid. We have reported SAR values in terms of W/g. In addition, temperature variations within the sample are expected to very small, in the initial heating process, and thus can be ignored [22].

## 3. Results and Discussions

### 3.1. XRD of Co_x_Mn_1-x_Fe_2_O_4_ (x = 0, 0.2, 0.4, 0.6, 0.8, and 1.0) Nanoparticles

**Figure 1:**
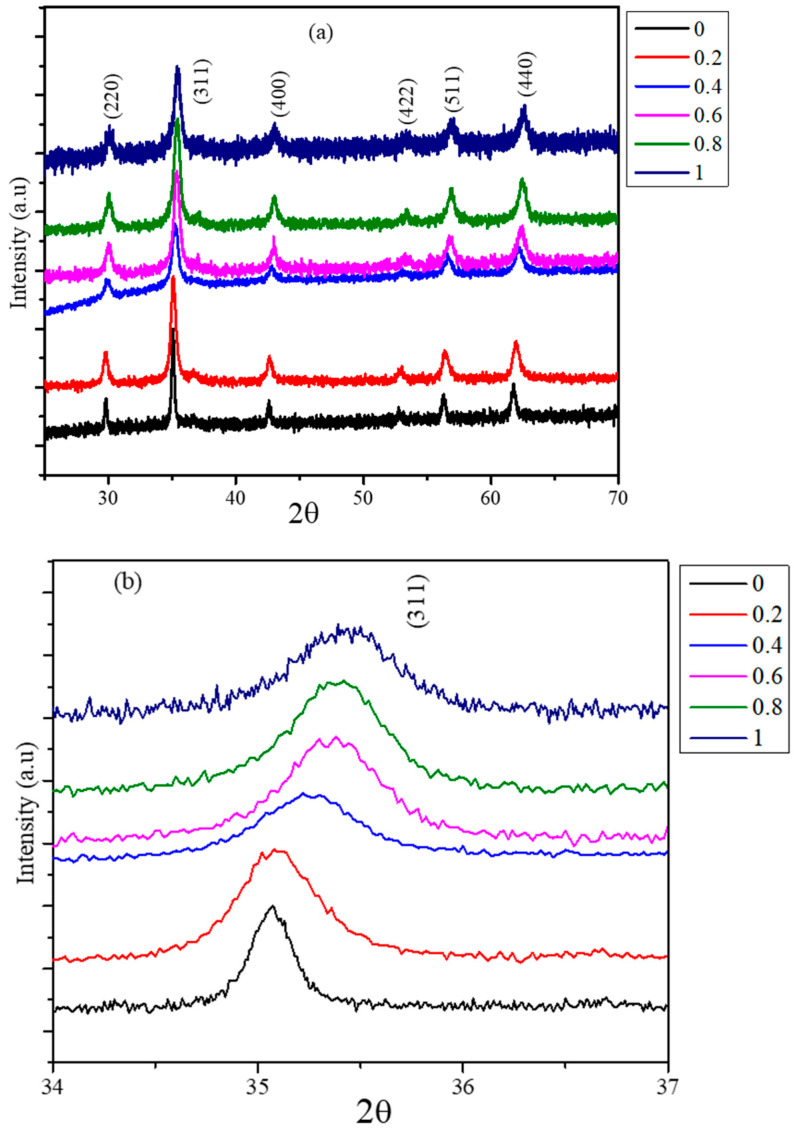
(**a**) X-ray diffraction patterns of Co_x_Mn_1-x_Fe_2_O_4_ (*x* = 0, 0.2, 0.4, 0.6, 0.8, and 1.0) nanoparticles. (**b**) Highest intensity peak (311) position shift with respect to cobalt concentration.

XRD patterns of the as synthesized Co_x_Mn_1-x_Fe_2_O_4_ (*x* = 0, 0.2, 0.4, 0.6, 0.8, and 1.0) nanoparticles are shown in Figure 1a. The XRD patterns display peaks corresponding to the spinel ferrite phase. In all the compositions, there are no additional peaks of other possible phases [23]. This indicates that the nanoparticles synthesized do not possess any phases of MnO_2_, CoO, and Fe_2_O_3_, which are expected because of the composition of the precursors used for the synthesis. The positions for all peaks have shifted to higher a diffraction angle from MnFe_2_O_4_ to CoFe_2_O_4_ as the cobalt concentration increases in the spinel. The shift in the position of highest intensity peak (311) with respect to concentration is shown in Figure 1b. The XRD patterns are used to obtain the lattice parameter and average crystallite sizes of the nanoparticles. The peaks corresponding to MnFe_2_O_4_ are narrow in width compared with CoFe_2_O_4_ peaks, indicating the average size of CoFe_2_O_4_ is considerably small in nature. The highest intensity peak (311) is fitted using Jade-XRD software to obtain the FWHM to determine the average crystallite sizes and the multiple peak fitting method is used to determine the lattice parameters. The average crystallite sizes, obtained using the Scherrer formula, show composition dependency. Composition-dependent lattice parameters and average crystallite sizes are listed in Table 1. The lattice parameters for MnFe_2_O_4_ (8.4889 Å) and CoFe_2_O_4_ (8.3891 Å) nanoparticles obtained agree with the reported values [24]. The lattice constant of mixed ferrite nanoparticles decreases as the concentration of Co^2+^ increases, which is expected as the ionic radii of the Co^2+^ are smaller than those of Mn^2+^. The average crystallite size of MnFe_2_O_4_ is 34.9 ± 0.6 nm, while it is 15.0 ± 0.3 nm for CoFe_2_O_4_. Though the conditions like pH, precursor concentrations, temperature, and reflux time used for the synthesis are identical for all the compositions, the average sizes obtained have strong dependency on the composition used for the synthesis. The nucleation and growth of the nanoparticles depend on the supersaturation and diffusion of the reactants used. The diffusion of the reactants to the growth site is controlled by the pH and ionic strength of the reaction mixture [25]. The observed difference in the particles sizes of MnFe_2_O_4_ and CoFe_2_O_4_ can be attributed to different diffusion rates of Co^2+^ and Mn^2+^ ions in the water medium. The average sizes of the mixed ferrite nanoparticles vary from 18.6 ± 0.5 nm to 16.6 ± 0.4 nm, and the average sizes decrease as the concentration of Co^2+^ increases in the precursor solution used for the synthesis.

### 3.2. TEM Images and SAED Pattern of Co_x_Mn_1-x_Fe_2_O_4_ (x = 0.0, 0.2, 0.4, 0.6, 0.8, and 1.0) Nanoparticles

**Figure 2:**
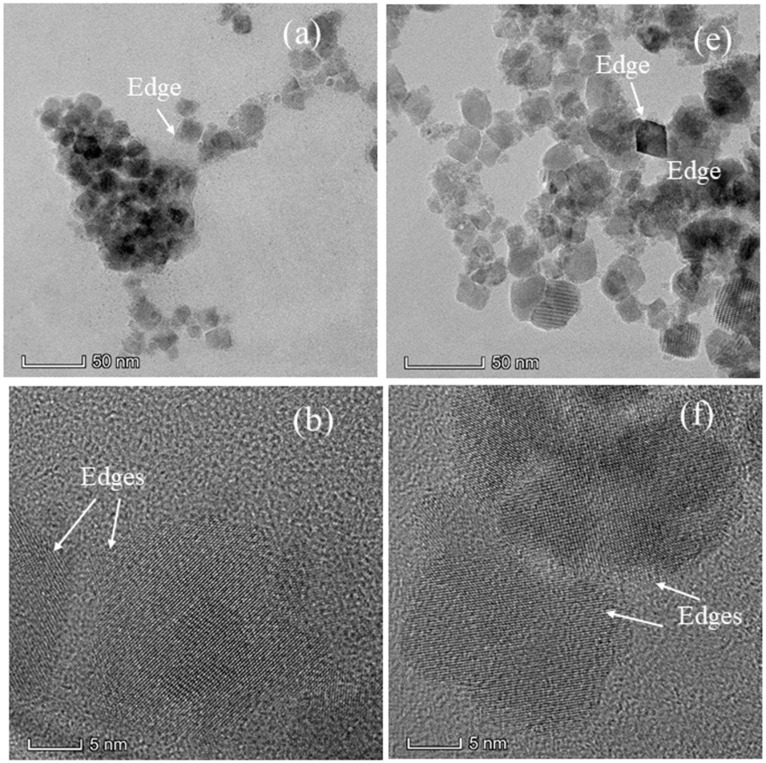

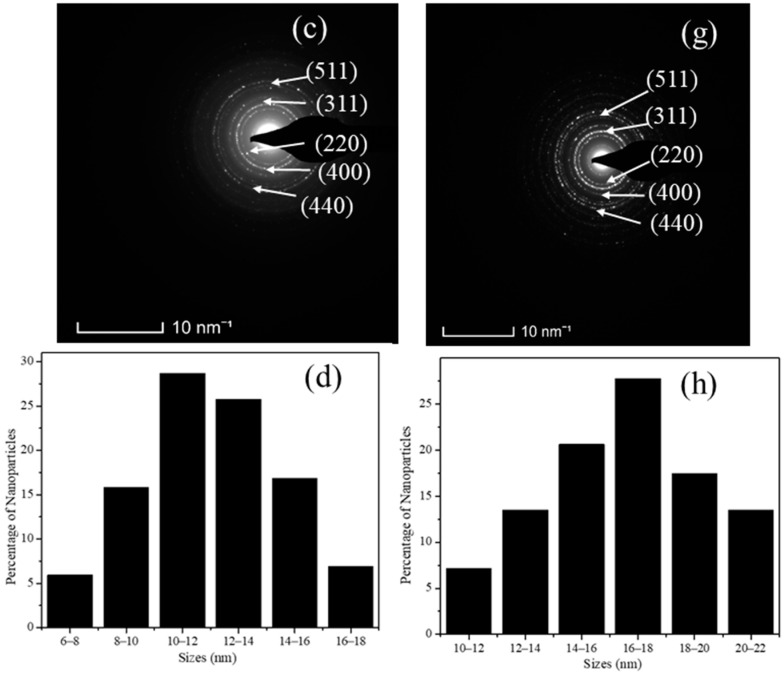
TEM bright field images, HRTEM, selected area electron diffraction patterns, and size distribution of (**a**–**d**) CoFe_2_O_4_ and (**e**–**h**) Co_0.2_Mn_0.8_Fe_2_O_4_ nanoparticles.

The structure and morphology of the nanoparticles synthesized iare further investigated using TEM bright field images and selected area electron diffraction (SAED). The as synthesized nanoparticles were dispersed in water and drop dried on the copper-coated TEM grid to obtain the bright field images. The bright field images, HRTEM image, diffraction patterns, and size distribution histograms of the nanoparticles with compositions Co_x_Mn_1-x_Fe_2_O_4_ (*x* = 0.2 and 1.0) are shown in Figure 2a–h. The nanoparticles synthesized from co-precipitation are non-spherical in shape with well-defined edges shown as an inset in HRTEM images. The SAED patterns shown in Figure 2c,g are indexed for ferrite spinel structure electron diffraction [26]. The absence of any diffraction rings corresponding to Fe_2_O_3_ phase indicates that the nanoparticles synthesized are pure ferrite phase, which is the reaffirmation of the purity of the phases observed from diffraction patterns. The size distributions of the nanoparticles are obtained using image J software; the nanoparticles with well separated boundary are considered for the measurement. The percentage of particles with respect to sizes is shown in Figure 2d,h. The size distributions of the nanoparticles are very broad; for *x* = 1.0, the sizes vary from 6 to 18 nm with a significant number of particles having sizes around 14 nm. In the case of *x* = 0.2 composition, the nanoparticles have averages sizes around 16–18 nm.

### 3.3. Raman Spectra of Co_x_Mn_1-x_Fe_2_O_4_ (x = 0.0, 0.2, 0.4, 0.6, 0.8, and 1.0) Nanoparticles

**Figure 3:**
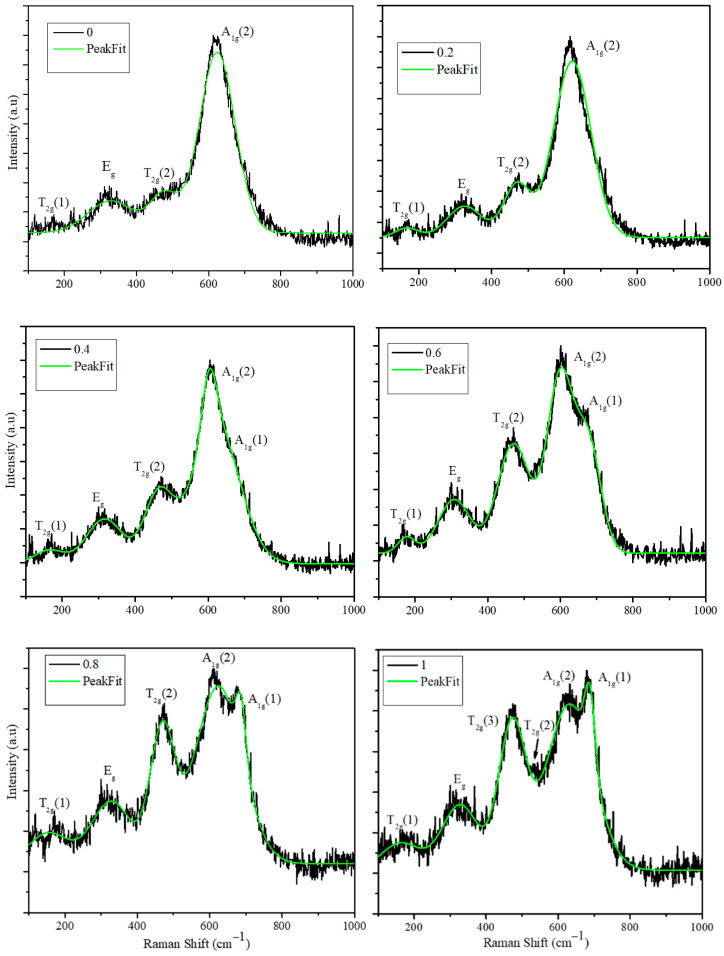
Raman spectra of Co_x_Mn_1-x_Fe_2_O_4_ (*x* = 0, 0.2, 0.4, 0.6, 0.8, and 1.0) nanoparticles (the composition and fitting of the peaks are marked in the figures; the green line indicates the peak fitting).

Raman spectra of the nanoparticles were obtained with the instrument equipped with the CCD detector and the excitation wavelength 532 nm produced by solid-state laser. The Raman spectra of the as synthesized nanoparticles are shown in Figure 3. The spectra of all the compositions (*x* = 0, 0.2, 0.4, 0.6, 0.8, and 1.0) are shown separately and peak fittings are shown as a green color solid line. The Raman data are analyzed for peak position and intensity, which depend on the site occupancy of octahedral and tetrahedral sites. MnFe_2_O_4_ has a normal spinel structure in which divalent Mn^2+^ ions occupy tetrahedral A sites, while octahedral B sites are occupied by trivalent cations (Fe^3+^) [27]. CoFe_2_O_4_ nanoparticles has an inverse spinel structure in which divalent Co^2+^ ions occupy half of the octahedral (B) sites and trivalent Fe^3+^ ions are distributed equally among A and B sites. The cubic crystal symmetry of the spinel structure has well defined Raman active vibrational modes. The group theory calculations predict the phonon distribution bands A_1g_+E_g_+ three T_2g_ for the cubic spinel structure [28]. Raman spectra of all the nanoparticles show the absence of a peak at 292 cm^−1^, indicating the absence of the Fe_2_O_3_ phase in the as synthesized nanoparticles [29]. The laser power used to record Raman spectra is optimized in such way that ferrite particles do not oxidize to form the Fe_2_O_3_ phase.

Raman spectra of the CoFe_2_O_4_ phase (*x* = 1.0) show characteristic peaks of inverse spinel; the bands at 684 and 633 cm^−1^ are assigned to the tetrahedral breathing modes of A_1g_(1) and A_1g_(2), respectively. A_1g_(1) and A_1g_(2) correspond to the symmetric stretching of oxygen atoms with respect to Fe and Co ions (Fe-O and Co-O bonds in tetrahedral sites). The intensity ratios of A_1g_(1) and A_1g_(2) peaks will provide the information about degree of inverse nature of the spinel structure. The asymmetric stretching (T_2g_(2)-Fe-O) and bending (T_2g_(3)-Fe(Co)-O) are assigned to the bands at 533 and 472 cm^−1^, respectively. The band at 326.7 cm^−1^ is assigned to the symmetric bending of Fe(Co)-O. The low intensity peak corresponding to the T_2g_ mode is assigned to the translation motion of the tetrahedron [30]. Raman spectra show inverse spinel structure of CoFe_2_O_4_ nanoparticles and rule out the presence of impurity phases like CoO and Fe_2_O_3_, which is in agreement with the XRD patterns obtained from the CoFe_2_O_4_ nanoparticles. The Raman spectra of MnFe_2_O_4_ (*x* = 0.0) are significantly different from the CoFe_2_O_4_ nanoparticles; the A_1g_ peak at 622 cm^−1^ is not split, indicating the symmetric stretching of Mn-O bond of tetrahedral site; furthermore, it has T_2g_(2) and E_g_ bands, which are assigned to the bending vibrational modes of Mn-O and Fe-O, respectively. The introduction of Co^2+^ into the lattice (*x* = 0.2, 0.4, 0.6, and 0.8) has led to the redistribution of cations (Co^2+^, Mn^2+^, and Fe^3+^). The corresponding spectra presented in Figure 3 show the splitting of the A_1g_ peak with composition *x* = 0.4 showing a significant split, which further increases with the increase of Co^2+^ ions in the mixed ferrite. The intensity ratios of the A_1g_(1) and A_1g_(2) are obtained and compared in Table 2. The cation redistribution can be seen from the intensity ratios provided in the table as more Fe^3+^ ions are transferred to tetrahedral sites, as is evident by the A_1g_ peak position and its subsequent shift to the higher wave number at 680 cm^−1^. The peak at 680 corresponds to the Fe-O bond stretching in the tetrahedral site. The intensity ratio of 1.05 and 0.98 for compositions *x* = 1.0 and 0.8 indicates that the Co^2+^ and Fe^3+^ are equally distributed in the tetrahedral sites. In the case of compositions *x =* 0.4 and 0.6, the intensity ratios are 0.75 and 0.84, which indicate that less tetrahedral sites are occupied by Fe^3+^. This is because of the presence of Mn^2+^ ions, which are preferred for the tetrahedral site owing to the high crystal field splitting energy stabilization. The Fe-O stretching is observed at 683 cm^−1^ (A_1g_ (1)) for *x* = 0.0, whereas it shifts to the lower wave number with increasing concentration of Mn^2+^, which can be attributed to the Fe(Co, Mn)-O stretching. The vibrational mode corresponding to the Co-O bond appears at 633 cm^−1^ and shifts to the lower number with increasing Mn^2+^ concentration. 

### 3.4. Mössbauer Spectrum of Co_0.2_Mn_0.8_Fe_2_O_4_ Nanoparticles

**Figure 4:**
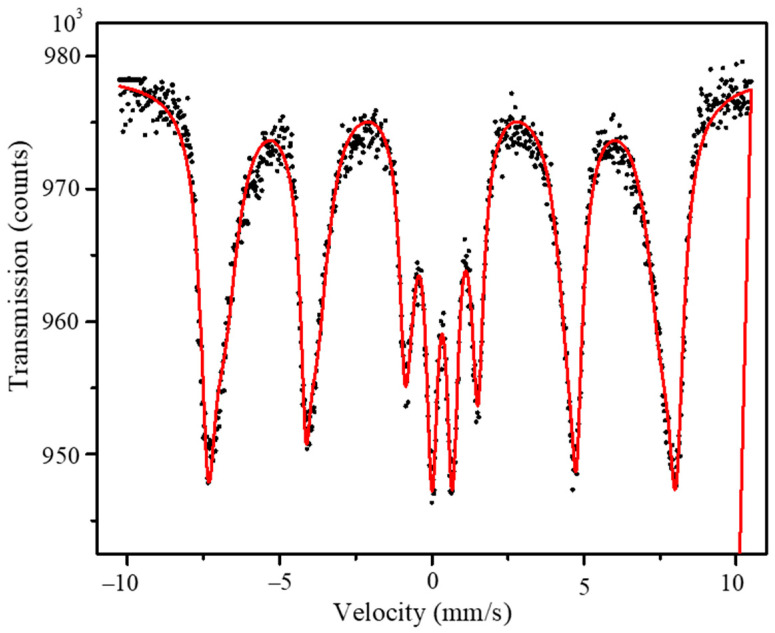
Room temperature Mössbauer spectrum of Co_0.2_Mn_0.8_Fe_2_O_4_. The dots represent the experimental data and the red solid line represents the fitting.

Figure 4 shows the Mössbauer spectrum of Co_0.2_Mn_0.8_Fe_2_O_4_ at room temperature and the fitting. The spectrum was fitted with the two magnetic sextets for Fe at the A and B sites and a doublet. The magnetic hyperfine parameters for the two magnetic sextets obtained from the fitting were the magnetic hyperfine field (H_hf_) = (44.62 ± 0.09), (47.78 ± 0.03) T; the quadrupole splitting (QS) = (−0.03 ± 0.01) mm/s, (0.006 ± 0.008) mm/s; and the isomer shift (IS) = (0.36 ± 0.01) mm/s, (0.32 ± 0.01) mm/s for the iron at A and B sites, respectively. The percentage of the doublet was 15% and it has QS = (0.67 ± 0.01) mm/s and IS = (0.34 ± 0.01) mm/s. This doublet represents the small nano-size particles in the superparamagnetic state, which is in agreement with previously reported observations by Noh et al. [31] for manganese ferrites. The values of the QS for the two magnetic sextets are almost zero, indicating the cubic symmetry with an inverse spinel structure, which is in agreement with Raman spectra data.

### 3.5. Magnetic Characterization of Co_x_Mn_1-x_Fe_2_O_4_ (x = 0.0, 0.2, 0.4, 0.6, 0.8, and 1.0) Nanoparticles

**Figure 5:**
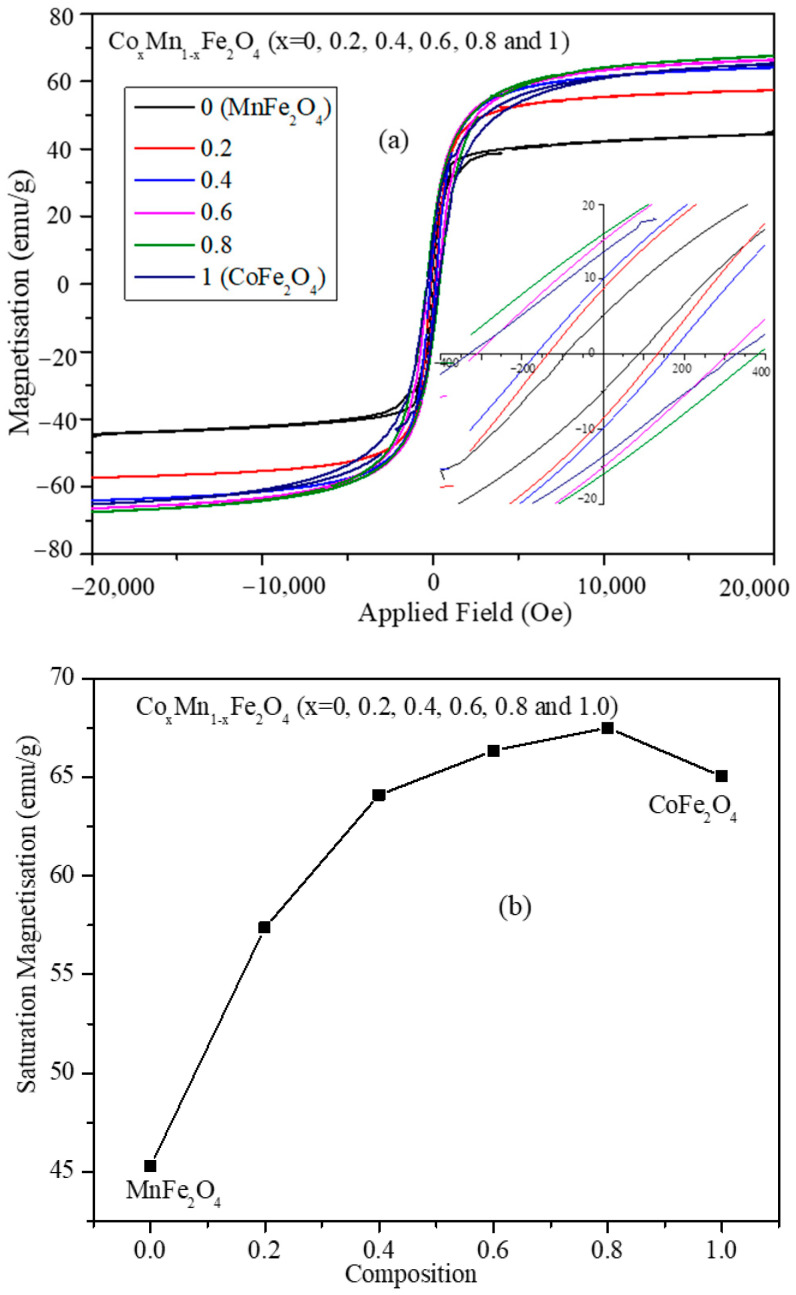
(**a**) Magnetic hysteresis loops obtained at room temperature in the field range of −2.0 T to +2.0 T for Co_x_Mn_1-x_Fe_2_O_4_ (*x* = 0.0, 0.2, 0.4, 0.6, 0.8, and 1.0) nanoparticles. (**b**) Mass normalized saturation magnetization (M_s_) value vs. Co^2+^ concentration of mixed ferrite (line in (**b**) is just a guide for the eye).

Magnetic hysteresis (MH) loops obtained for the mixed ferrite nanoparticles are shown in Figure 5a. The magnetic hysteresis loops were obtained at room temperature by applying a magnetic field in the range of −2.0 T to +2.0 T. The MH plots show that the nanoparticles possess a significant coercive field (H_c_) and remnant magnetization (M_r_) at room temperature; the values for all the compositions are listed in Table 3. These values show high composition dependency; the nanoparticles of composition Co_0.2_Mn_0.8_Fe_2_O_4_ have the highest remanent magnetization (16.05 emu/g) and coercive field (382.6 Oe). For the other compositions (*x* = 0.4, 0.6, and 0.8) of mixed ferrite nanoparticles, remanent magnetization and coercive field decrease with the increase of cobalt concentration. The CoFe_2_O_4_ nanoparticles possess the least remanent magnetization and coercive filed of 5.07 emu/g and 90 Oe, respectively. The saturation magnetization values (Figure 5b) show a non-monotonic behavior as a function of composition. This can be attributed to the change in site occupancy of cations in tetrahedral and octahedral positions as indicated by the Raman spectra. The trends of the remanent magnetization and coercivity values obtained from the hysteresis loops of Figure 3a can be attributed to the average crystallite sizes of the Co_x_Mn_1-x_Fe_2_O_4_ (*x* = 0.0, 0.2, 0.4, 0.6, 0.8, and 1.0) nanoparticles. The average crystallite sizes of the nanoparticles have similar trends to those of the remanent magnetization and coercive field.

**Figure 6:**
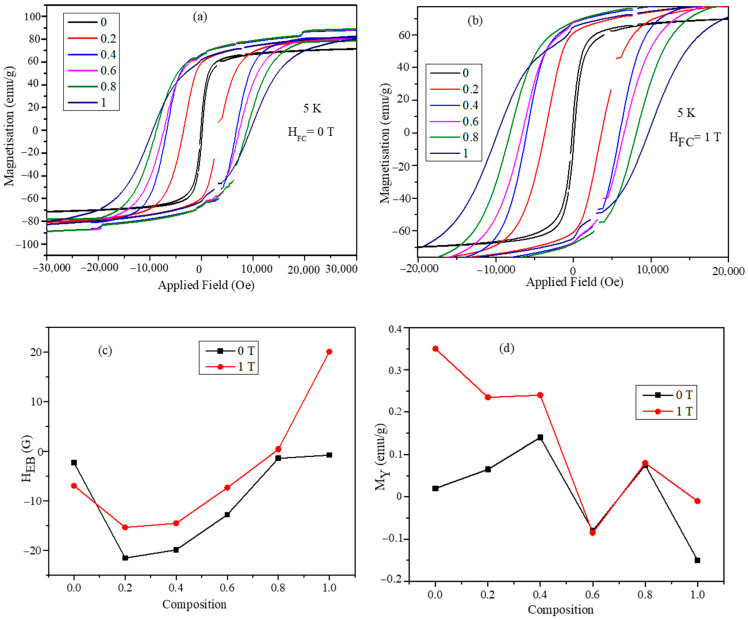
The magnetic hysteresis loops obtained at temperature 5 K (**a**) under zero field cooled (ZFC) condition and (**b**) under 1 T field cooled condition. (**c**) The exchange bias field as a function of composition at temperature 5 K under several field cooled values 0 and 1 T. (**d**) The vertical hysteresis loop shifts as a function of temperature at ZFC and 1 T field cooled conditions (lines in (**c**,**d**) are just guides for the eye).

The low temperature MH plots obtained at 5 K with zero field cooled and 1 T applied field cooled conditions are shown in Figure 6a,b. The exchange bias values of the mixed ferrite nanoparticles were obtained from these hysteresis loops. The horizontal shift in the hysteresis loops was defined as the exchange bias field, H_EB_. The exchange bias field, H_EB_, was calculated using the following formula [32]:

(8) $$H_{\mathrm{EB}}=\frac{H_{C1}+H_{C2}}{2}$$

Here, the coercive field at the descending branch of the hysteresis loop is $H_{C1}$, and that on the ascending branch is $H_{C2}$.

The vertical shift in the hysteresis loops was calculated using the following formula:

(9) $$M_{y}=\frac{M_{R1}+M_{R2}}{2}$$

Here, the remnant magnetization value at the descending branch of the hysteresis loop is $M_{R1}$ and the one on the ascending branch is ${\;M}_{R2}$.

The exchange bias plots obtained at 5 K temperature for both H_EB_ and M_Y_ are shown in Figure 6c,d. The H_EB_ values decrease with the increase in the Co^2+^ concentration, and it is at a minimum for the composition with *x* = 0.2. The absolute values of H_EB_ are always higher for the 1 T field conditions compared with the zero field conditions. The H_EB_ values are negative except for CoFe_2_O_4_ nanoparticles under 1 T cooled condition. The vertical exchange biases M_Y_ obtained at 5 K also show composition dependency with non-monotonic behavior. All the compositions have positive exchange bias under zero and 1 T field cooled conditions, except the composition *x* = 0.6, which has negative exchange bias. The coercive field values obtained from the MH plots of Figure 6a,b are listed in Table 4. MnFe_2_O_4_ nanoparticles have the lowest coercive field of 142.68 G and 138.45 G under both zero and 1 T field conditions, respectively. The coercivity values increase with the increase in the cobalt concentration of the mixed ferrite nanoparticles. CoFe_2_O_4_ nanoparticles possess the highest coercive field of 9716.8 and 9461.25 under zero and 1 T field cooled fields, respectively. The absolute values of the coercive field are slightly higher for the zero field compared with 1 T field cooled for all the compositions. The coercive filed values obtained at room temperature have a different trend with respect to compositions compared with the values obtained at room temperature, as listed in Table 3. The exchange bias values obtained at 5 K temperature are bit low to have a significant effect on the Neel and Brownian relaxation times.

### 3.6. Magnetic Hyperthermia Studies of the Co_x_Mn_1-x_Fe_2_O_4_ (x = 0.0, 0.2, 0.4, 0.6, 0.8, and 1.0) Nanoparticle Water Dispersions

Magnetic hyperthermia efficiency of the PEG-coated mixed ferrite nanoparticles was obtained using a nanoscale Bio magnetics instrument. Heating profile curves were obtained for the Co_x_Mn_1-x_Fe_2_O_4_ (*x* = 0.0, 0.2, 0.4, 0.6, 0.8, and 1.0) nanoparticle concentrations of 3, 5, 7, and 10 mg/mL at frequencies of 765.85, 634.45, 491.10, 390.25, 349.20, and 333.5 kHz and field strengths of 200, 250, 300, and 350 G. The heating profiles of PEG-coated CoFe_2_O_4_ and Co_0.2_Mn_0.8_Fe_2_O_4_ nanoparticles are shown in Figure 6a–d. To study the effect of field frequency and strength on the heating ability of the nanoparticles, heating profiles are obtained by keeping field strength constant at 350 G, while the frequency was varied between 333.5 and 765.86 kHz. To study the effect of field strength on the heating ability, the frequency was set at 765.95 kHz and the field strength was varied between 200 and 350 G. These instrument parameters are well within the permissible levels of *C* = *H* × *f* = 5 × 10^9^ Am^−1^s^−1^ (6.25 × 107 Oe Hz) for use with human trails. Heating profiles were recorded for a given concentration and field parameters until the temperature of the nanoparticle dispersion reached 70 °C [33]. The readings were taken for a maximum of 20 min exposure time when the dispersion temperature did not exceed 70 °C. The heating profiles clearly demonstrate that particle concentration, strength, and frequency of the AMF field and composition of the nanoparticle dispersion all have a significant impact on magneto thermic ability. The heating profiles are obtained using identical conditions and SAR values were determined using the initial slope of the heating curve using Equation (7).

**Figure 7:**
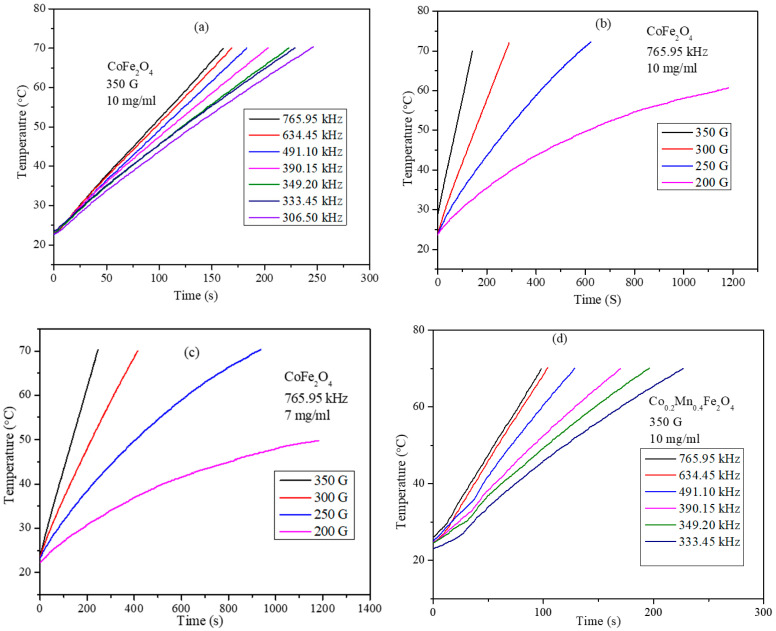
Heating profiles of PEG-coated CoFe_2_O_4_ nanoparticles: (**a**) at 350 G for 10 mg/mL concentration, (**b**) at 765.95 kHz for 10 mg/mL concentration, (**c**) at 765.95 kHz for 7 mg/mL concentration, and (**d**) PEG-coated Co_0.2_Mn_0.8_Fe_2_O_4_ at 350 G for 10 mg/mL concentration.

**Figure 8:**
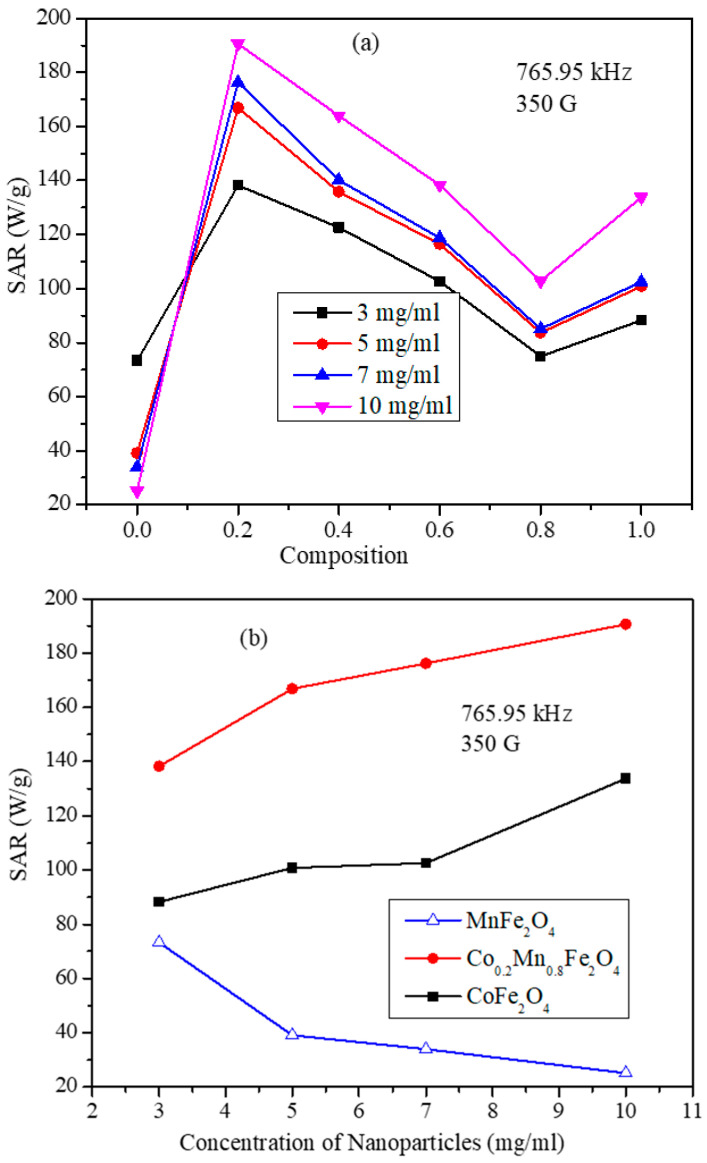
(**a**) SAR values as a function of the compositions Co_x_Mn_1-x_Fe_2_O_4_ (*x* = 0, 0.2, 0.4, 0.6, 0.8, and 1.0) nanoparticle with concentrations of 3, 5, 7, and 10 mg/mL at 765.95 kHz frequency and 350 G field strength. (**b**) Concentration dependent SAR of MnFe_2_O_4_, CoFe_2_O_4_, and Co_0.2_Mn_0.8_Fe_2_O_4_ nanoparticles at 765.95 kHz frequency and 350 G field strength (lines are just guides for the eye).

The heating profile curves displayed in Figure 7 show that, at very low frequency and field strength, the nanoparticles do not increase the temperature above 44 °C, which is the crucial requirement for the hyperthermia, such a combination of low frequency and field parameters were not used in the measurement. The heating profiles for all the compositions of Co_x_Mn_1-x_Fe_2_O_4_ nanoparticle were obtained under similar conditions and SAR values for the concentrations of 3, 5, 7, and 10 mg/mL obtained from the heating profiles are shown in Figure 8a. The SAR values of the nanoparticles show strong dependency on the composition of the nanoparticles. The SAR values obtained for the 10 mg/mL concentration are 765.95 kHz and 350 G field strength are 25.07, 190.61, 163.94, 138.37, 102.76, and 133.74 W/g for compositions *x* = 0, 0.2, 0.4, 0.6, 0.8, and 1, respectively. Although, the expected trend of SAR values might be expected to show a similar trend to that of the saturation magnetization (shown in Figure 5b). However, interestingly, the composition with *x* = 0.8, which has highest saturation magnetization value of 67.63 emu/g, displayed the lowest SAR values at all concentrations among mixed ferrite nanoparticles. For each concentration, the SAR value is highest for the nanoparticles with composition x = 0.2 and with saturation magnetization of 57.41 emu/g. The maximum SAR value obtained is 190.61 W/g for the sample with composition *x* = 0.2 and particle concentration of 10 mg/mL. The SAR values decreased with the further increase in Co^2+^ composition, reaching a minimum for *x* = 0.8, and then increased slightly for the CoFe_2_O_4_ (*x* = 1.0) nanoparticles. This non-linear behavior of the SAR with respect to saturation magnetization of the nanoparticles can be attributed to several factors, such as the relaxation times (Neel and Brownian relaxation), morphology, size, and size distribution. The Neel relaxation given by Equation (6) has a strong dependency on the magnetic anisotropy constant (K), which again depends on the coupling interaction of cations occupied in tetrahedral and octahedral sites through oxygen [34]. The optimum site occupancy of Co^2+^ in the tetrahedral and octahedral sites is required. The remnant magnetization values listed in Table 3 possess a trend similar to that of SAR of the mixed ferrite nanoparticles, except for the pure CoFe_2_O_4_ nanoparticles. The concentration-dependent SAR values of MnFe_2_O_4_, CoFe_2_O_4_, and Co_0.2_Mn_0.8_Fe_2_O_4_ nanoparticles are shown in Figure 8b. The SAR values of MnFe_2_O_4_ nanoparticles decrease non-linearly with the increase in the concentration of nanoparticle dispersion. Interestingly, for the CoFe_2_O_4_ and mixed ferrite nanoparticles, the SAR values increase with the increase in the concentration of the particle dispersions used for the measurements.

**Figure 9:**
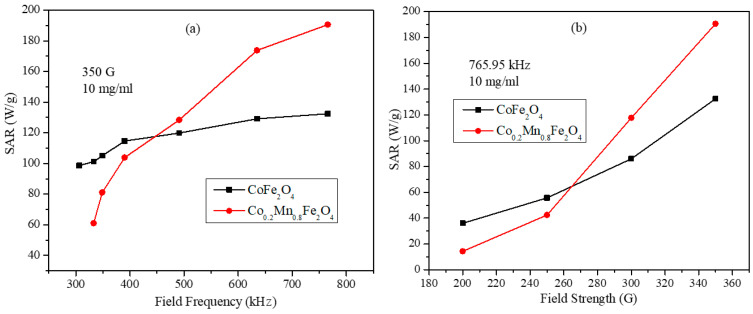
(**a**) SAR values as a function of alternating magnetic field (AMF) frequency at field strength of 350 G for 10 mg/mL of CoFe_2_O_4_ and Co_0.2_Mn_0.8_Fe_2_O_4_ nanoparticles and (**b**) SAR values as a function of field strength at fixed AMF frequency of 765.95 kHz for 10 mg/mL of CoFe_2_O_4_ and Co_0.2_Mn_0.8_Fe_2_O_4_ nanoparticles (lines are just guides for the eye).

As shown in Figure 8b, the nanoparticles with composition Co_0.2_Mn_0.8_Fe_2_O_4_ possess the maximum SAR value (190.61 W/g for 10 mg/mL concentration at 765.95 kHz and 350 G). These particle dispersions were examined further for frequency and field strength dependency along with CoFe_2_O_4_ nanoparticles. The frequency and field strength dependent SAR values for the samples Co_0.2_Mn_0.8_Fe_2_O_4_ and CoFe_2_O_4_ are shown in Figure 9a,b. The SAR values were obtained for 10 mg/mL concentration at fixed field strength of 350 G with variable field frequencies and at fixed frequency of 765.95 kHz with variable field strengths. The frequency dependent SAR values of the CoFe_2_O_4_ nanoparticles show a roughly linear behavior. On the other hand, the SAR values of the Co_0.2_Mn_0.8_Fe_2_O_4_ nanoparticles show a non-linear dependency on the frequency of the AMF. This behavior is different from the linear behavior suggested by the linear response theory, which hints at the role of inter-particle interactions [35]. At the low frequencies of 390.15, 349.20, and 333.45 kHz, the SAR values of the Co_0.2_Mn_0.8_Fe_2_O_4_ sample are smaller than those of the CoFe_2_O_4_ nanoparticles. However, the SAR values for the Co_0.2_Mn_0.8_Fe_2_O_4_ nanoparticles increased rapidly at higher frequencies and became considerably larger than those of CoFe_2_O_4_ nanoparticles. At a fixed frequency, the SAR values of Co_0.2_Mn_0.8_Fe_2_O_4_ are higher than the SAR values of the CoFe_2_O_4_ nanoparticles at the high fields of 300 and 350 G, whereas they are lower at the low field strengths of 200 and 250 G. Interestingly, both samples do not display the quadratic field dependence as suggested by the linear response theory. These deviations can be attributed to the inter-particle interactions and the wide size distributions.

### 3.7. Agar Hydrogel Phantom for the Hyperthermia Measurements

Agar hydrogel phantom, which is commonly used in MRI studies of contrast agents, was employed for the magnetic hyperthermia measurements of CoFe_2_O_4_ and Co_0.2_Mn_0.8_Fe_2_O_4_ nanoparticles [36]. Hydrogels are three-dimensional polymer networks with tissue-mimicking properties and the ability to maintain a significant amount of water in their swollen state. The agarose gel dissolves in water and forms a transparent and mechanically stable hydrogel in which the pH of the gel is maintained neutral [33]. Kaczmarek et al. have reported the hyperthermia studies on the effect of tissue-mimicking phantom compressibility on the effectiveness of magnetic hyperthermia of agar phantoms. They have shown that single and cluster nanoparticles with different concentration of agar possess variable thermal heating. SAR values proved that tissue-mimicking phantom compressibility affects magnetic losses in the AMF. The lower compressibility of agar gel showed lower thermal heating [37].

**Figure 10:**
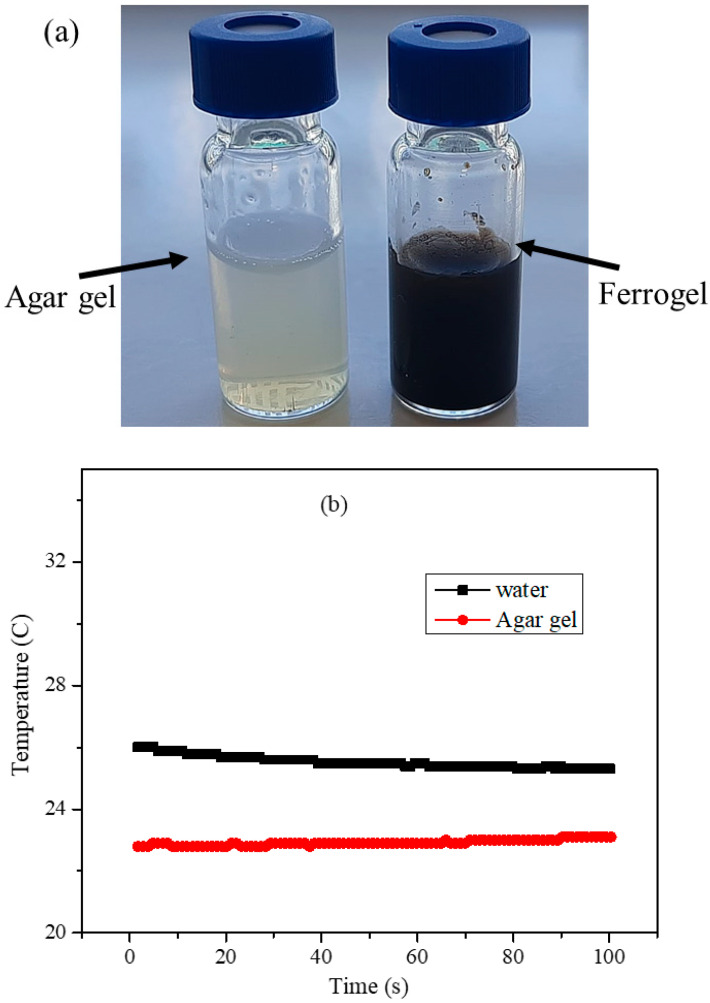
(**a**) Images of agar gel and ferrogel of the nanoparticles used for hyperthermia measurements. (**b**) Heating profiles of 1 mL of distilled water and agar gel.

Here, 50 mg of the agarose powder was added to the 1 mL of distilled water and, to this mixture, 10 mg of PEG-coated nanoparticle was added and sonicated for 10 min. The dispersion mixture was heated to 95 °C using a water bath; upon heating for 20 min, agar dissolves in water completely and forms homogenous solution at 95 °C and, upon cooling, it forms a homogenous ferrogel. The ager hydrogel and agar-ferrite magnetic ferrogel formed upon cooling are shown in Figure 10a. From the images, it can be observed that agar gel is transparent and the nanoparticles are uniformly distributed in agar gel. The heating profiles of pure water and agar gel, shown in Figure 10b, indicate that the water and agar gel do not cause any magnetic heating upon exposure to AMF. The heating measurements were carried out with different initial temperatures.

**Figure 11:**
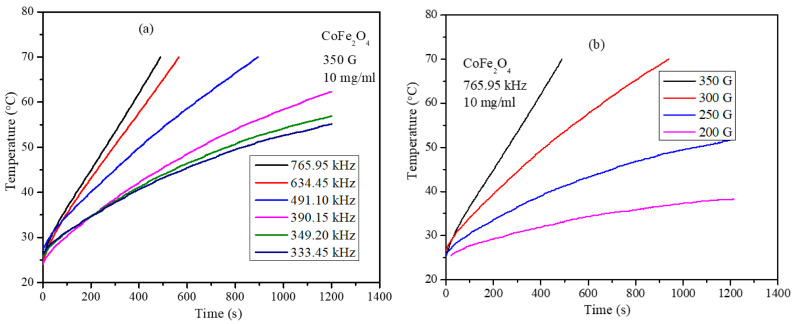

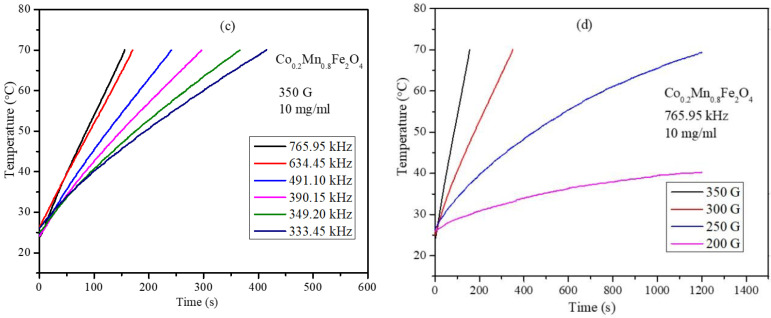
Heating profiles of PEG-coated 10 mg/mL CoFe_2_O_4_ nanoparticles and agar ferrogel: (**a**) at 350 G as a function of frequency and (**b**) at 765.95 kHz as a function of field strength. Heating profiles of PEG-coated 10 mg/mL Co_0.2_Mn_0.8_Fe_2_O_4_ nanoparticles and agar ferrogel: (**c**) at 350 G fixed field strength and as a function of frequency and (**d**) at 765.95 kHz fixed frequency and as a function of field strength.

The heating profiles obtained for 10 mg/mL nanoparticle concentration ferrogel of CoFe_2_O_4_ and Co_0.2_Mn_0.8_Fe_2_O4 nanoparticles at frequencies of 765.95, 634.45, 491.10, 390.15, 349.20, and 333.45 kHz and field amplitudes of 200, 250, 300, and 350 G are shown in Figure 11. The heating profiles show lesser heating compared with those obtained for PEG-coated nanoparticles dispersions in pure water.

**Figure 12:**
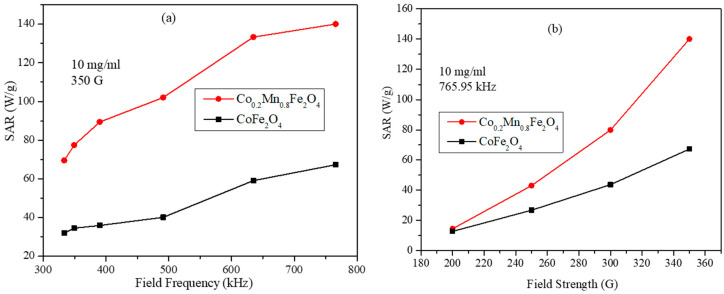
(**a**) SAR values as a function of AMF frequency at field strength of 350 G for 10 mg/mL of CoFe_2_O_4_ and Co_0.2_Mn_0.8_Fe_2_O_4_ nanoparticles and agar ferrogel. (**b**) SAR values as a function of field strength at fixed AMF frequency of 756.95 kHz for 10 mg/mL of CoFe_2_O_4_ and Co_0.2_Mn_0.8_Fe_2_O_4_ nanoparticles and agar ferrogel (lines are just guides for the eye).

The SAR values obtained from the heating profiles in Figure 11 for the CoFe_2_O_4_ and Co_0.2_Mn_0.8_Fe_2_O_4_ ferrogel are shown in Figure 12a,b. We can see that the SAR values for the ferrogel are lower than the SAR values of the water-dispersed nanoparticles (with the equal concentration) obtained under the same frequencies and field strengths. Interestingly, the trends of the SAR values are different compared with water particle dispersions, which can be observed from the frequency dependent plots shown in Figure 9a and Figure 12a. In Figure 12a, the SAR values for both samples show almost linear dependency on the frequency, with higher values for the Co_0.2_Mn_0.8_Fe_2_O_4_ ferrogel than those for CoFe_2_O_4_. The field strength dependent values displayed in Figure 12b show a sublinear dependency. Hence, the SAR results in Figure 12 are close to those expected by the LRT. This behavior can be attributed to the smaller inter-particle interactions in the ferrogel samples. The difference in the SAR values observed for the ferrogel and water dispersions can be attributed to the suppressed particle rotation, and thus the increased Brownian relaxation time given by Equation (5), which has a dependency on the viscosity of the medium. This leads to the suppression of the Brownian relaxation contribution in the effective relaxation time. Nevertheless, the SAR values obtained for the ferrogel are reasonably high and the particles possess significantly high heating ability for the tissue mimicking agar gel. These studies will be useful in designing the mixed ferrite based high efficiency nanoparticles for the hyperthermia applications in treating cancer both in vitro and in vivo.

## 4. Conclusions

Co^2+^ and Mn^2+^ divalent mixed ferrite nanoparticles with compositions Co_x_Mn_1-x_Fe_2_O_4_ (*x* = 0.0, 0.2, 0.4, 0.6, 0.8, and 1.0) are synthesized using a simple co-precipitation method. The structural and morphological properties of the nanoparticles were obtained using XRD, TEM, Raman spectroscopy, and Mössbauer spectroscopy. The MnFe_2_O_4_ nanoparticle has an average crystallite size of ~35 nm, while it is ~15 nm for CoFe_2_O_4_, with nanoparticles sizes decreasing with the Co^2+^ concentration of the mixed ferrite. The Raman data show that the MnFe_2_O_4_ nanoparticles have a normal spinel structure, and introduction of Co^2+^ causes the redistribution of Fe^3+^ ions among tetrahedral and octahedral sites. The splitting of the A_1g_ peak at 620 cm^−1^ indicates the redistribution of Co^2+^ and Fe^3+^ ions. The intensity ratio of the split peak A_1g_(1) and A_1g_(2) peak indicates significant redistribution of Co^2+^ and Fe^3+^ cations among tetrahedral and octahedral sites in mixed ferrite. Magnetic hysterics loops show that all the particles possess some remnant magnetization and coercivity. The saturation magnetization and the SAR values were found to display a nonmonotonic behavior as a function of composition. The saturation magnetization is highest for the *x* = 0.8 batch of particles (67.63 emu/g), while it is 65.19 emu/g for CoFe_2_O_4_. Heating profiles with frequencies of 765.95, 634.45, 491.10, 390.15, 349.20, and 333.45 kHz and field amplitudes of 200, 250, 300, and 350 G were obtained. The nanoparticle composition with *x* = 0.2 (Co_0.2_Mn_0.8_Fe_2_O_4_) with a saturation magnetization of 57.41 emu/g showed the highest SAR value of 190.61 W/g for 10 mg/mL concentration at a frequency of 765.95 kHz and 350 G field strength. The SAR values for the mixed ferrite and CoFe_2_O_4_ nanoparticles were found to increase with concentration, while in the case of MnFe_2_O_4_ nanoparticle dispersion, the SAR values decreased with concentration. Nanoparticles ferrogel were formed with composition *x* = 0.2 and CoFe_2_O_4_ with SAR values of 140.35 and 67.60 W/g, respectively. The heating efficiency of the mixed ferrite nanoparticles was found to depend on the site occupancy, particle size, concentration of magnetic dispersions, and viscosity of the surrounding medium. The SAR value obtained for the ferrogel is still high and the particles possess significantly high heating ability for the tissue mimicking agar gel. These findings will be helpful in engineering high efficiency heating mixed ferrite nanoparticles by optimizing the composition in the mixed ferrite nanoparticles for the magnetic hyperthermia applications.

## Figures and Tables

**Table 1:** The average crystallite sizes and lattice parameters of the Co_x_Mn_1-x_Fe_2_O_4_ (*x* = 0, 0.2, 0.4, 0.6, 0.8, and 1.0) nanoparticles.

Composition	Average Sizes (nm)	Lattice Constant (Å)
0 (MnFe_2_O_4_)	34.9 ± 0.6	8.4889
0.2	18.6 ± 0.5	8.4824
0.4	17.2 ± 0.3	8.4473
0.6	16.8 ± 0.4	8.4183
0.8	16.6 ± 0.4	8.4054
1(CoFe_2_O_4_)	15.0 ± 0.3	8.3891

**Table 2:** The peak positions and intensity ratios of Co_x_Mn_1-x_Fe_2_O_4_ (*x* = 0.0, 0.2, 0.4, 0.6, 0.8, and 1.0) nanoparticles.

Composition	A_1g_(1)	A_1g_(2)	T_2g_(1)	E_g_	T_2g_(3)	I_A1g_(1)/I_A1g_(2)
0	622		480	324	166	
0.2	619		471	327	166	
0.4	664	606	473	318	167	0.75
0.6	669	604	471	308	176	0.84
0.8	680	625	471	328	177	0.98
1	683	633	472	327	180	1.05

**Table 3:** The coercive field and remnant magnetization of the nanoparticles obtained at room temperature.

Composition	Coercive Field (Oe)	Remnant Magnetization (Emu/g)
0 (MnFe_2_O_4_)	329.00	13.66
0.2	382.60	16.05
0.4	308.80	15.11
0.6	165.10	12.49
0.8	135.45	8.62
1 (CoFe_2_O_4_)	90.00	5.07

**Table 4:** The Coercive field values of the nanoparticles obtained with zero and 1 T field cooled conditions at 5 K temperature.

Composition	Coercive Field (G)	
	Zero Field Cooled	1 T Field Cooled
0 (MnFe_2_O_4_)	142.68	138.45
0.2	3471.55	3661.55
0.4	6715.60	6028.35
0.6	7258.10	6566.45
0.8	8748.60	8147.20
1(CoFe_2_O_4_)	9716.80	9461.25

## Data Availability

Not applicable.
